# Investigation of particle penetration in a nasal maxillary ostium with optimised T-junction geometry and pulsatile flow

**DOI:** 10.1007/s10237-025-02015-9

**Published:** 2025-10-06

**Authors:** Patrick Warfield-McAlpine, David F Fletcher, Kiao Inthavong

**Affiliations:** 1 Department of Mechanical Manufacturing Mechatronic Engineering, RMIT University, PO Box 71, Bundoora, 3083 Australia; 2 School of Chemical and Biomolecular Engineering, The University of Sydney, Sydney, NSW 2006 Australia

**Keywords:** Aerosol, Particle deposition, Drug delivery, Pulsating flow, CFD, Laminar, T-junction

## Abstract

Effective drug delivery to the maxillary sinus is often limited by the narrow and variable shape of the maxillary ostium. To better understand and predict how surgical changes affect drug transport, the ostium can be modelled as a simplified T-junction. The geometric configuration of these junctions plays a crucial role in managing particle flow; however, optimal design parameters remain under-explored. This paper addresses this gap, by simulating a range of radius of curvatures $R_{\text {c}}$ at the T-junction and oscillatory flows with pulsation frequencies of 0, 30, 45, 60 and 75 Hz to analyse their effects on particle penetration and distribution. The results revealed that an anterior $R_{\text {c}}$ enhanced particle outflow through the *y*-branch (perpendicular) outlet, while a posterior $R_{\text {c}}$ limited this outflow. Comparisons of pulsating frequencies further showed that a lower frequency improved penetration into the *y*-branch. Interestingly, applying both anterior and posterior $R_{\text {c}}$ did not yield better performance than an anterior $R_{\text {c}}$ alone. Furthermore, a constant flow rate where $f = 0$ Hz promoted greater particle outflow through the *y*-branch in the T-junction model. However, a pulsating frequency of 30 Hz improved deposition in the nasal airway. The study underscores the potential of targeted geometric adjustments to optimise flow and deposition in the maxillary ostium, providing valuable insight into drug delivery strategies and inhalation toxicology.

**Supplementary Information:**

The online version contains supplementary material available at 10.1007/s10237-025-02015-9.

## Introduction

T-junctions are fundamental components in fluid systems, facilitating the management of flow distribution and direction. Their applications span a wide range of industries, including industrial piping networks and chemical processing, as well as biomedical fields such as respiratory and cardiovascular health, and drug delivery systems (Baker et al. 2007; Ejaz et al. 2022; Takeuchi and Karino 2010; Karino et al. 1979; Li et al. 2019; Khamooshi et al. 2022). The functionality of T-junctions is primarily determined by their geometric configuration, which allows for either the convergence or divergence of fluid streams. In typical scenarios, two streams converge into a single outlet or a single stream diverges into two perpendicular outlets; however, the present study focuses on the divergence of flow, where a single inlet bifurcates into two outlets; one perpendicular and the other axial as an analogue to a maxillary ostium.

The narrow, laterally positioned maxillary ostium presents a significant anatomical barrier to effective drug penetration into the maxillary sinus. The ostium is typically characterised as an elongated cylindrical conduit linking the nasal airway to the maxillary sinus (Pourmehran et al. 2025; Siu et al. 2021; Inthavong et al. 2020; Pourmehran et al. 2020; Hood et al. 2009). This structure can be idealised using a T-junction model to systematically investigate the impact of anatomical variations. This modelling strategy aligns with the simplified rectangular plane and T-junction configuration employed by Hood et al. (2009).

The maxillary sinuses, the largest among the paranasal sinuses, play a critical role in producing mucus that lubricates the nasal passages and conditions inspired air to facilitate efficient pulmonary gas exchange. Inflammatory processes within the maxillary sinus can obstruct the ostia, the primary drainage pathways of the paranasal sinuses, thereby compromising mucociliary clearance. Management of such obstructions typically involves nasally delivered drug therapy (Pourmehran et al. 2025; Vahaji et al. 2021).

The maxillary sinus connects to the nasal airway through the ostium, a small tubular passage typically 1-5 mm in diameter and 6 mm in length (Aust and Drettner 1974). If drug therapy fails to restore physiological drainage, surgery is required to widen the ostium and improve airflow. Functional Endoscopic Sinus Surgery (FESS) enhances sinus ventilation and mucociliary clearance by resecting obstructive tissue to enlarge the maxillary ostium (Wofford et al. 2015). This increases the pathway for fluid transport, though the optimal enlargement varies due to anatomical differences, necessitating patient-specific planning using medical imaging. Alongside surgery, patients often continue topical drug therapy to treat inflamed sinus mucosa. Optimising airflow within the nasal cavity is essential for effective drug delivery and improved treatment outcomes. However, poor ventilation and restricted access to the ostia make drug delivery challenging.

The maxillary sinus penetration of a nasal saline rinse using blue dye was evaluated in 17 patients with varying ostium sizes (Grobler et al. 2008). The study showed that ostia smaller than 1.26 mm did not allow dye penetration, while a minimum diameter of 3.95 mm was needed to achieve a 95% chance of successful delivery. Given that ostium sizes typically range from 1 to 5 mm in diameter, these findings indicate that nasal rinses may be ineffective for patients with smaller ostia, who could benefit from ostium enlargement before continuing post-surgical drug treatments.

Computational fluid dynamics (CFD) is widely used to study drug deposition in the maxillary sinus by simulating airflow and particle transport in anatomically accurate models (Wofford et al. 2015; Vahaji et al. 2021; Pourmehran et al. 2020, 2025). CFD helps identify how variations in ostium size and shape affect medication delivery efficiency and enables the optimisation of drug delivery devices and techniques. Additionally, patient-specific CFD models based on medical imaging can support personalised treatment planning and improve surgical outcomes.

Pulsating flow has been shown to enhance mass transfer in T-junctions, particularly under laminar and transitional flow conditions. Zaki et al. (2011) demonstrated that pulsating flow can improve mass transfer rates significantly, achieving enhancements between 1.2 and 5.5 times compared with steady laminar flow (Mohan et al. 2014). This is attributed to the periodic fluctuations in flow velocity, which promote additional mixing. In contrast, under steady turbulent flow regimes, the influence of pulsating flow is minimal, as the turbulence generated by the flow overshadows the effects of pulsations (Zaki et al. 2011).

Nebulisers are commonly used to deliver aerosolised particles to the nasal cavity. Such devices use acoustic or pulsating frequencies to improve deposition and minimise drug loss. Historically, the delivery of medication to the sinuses have adopted frequencies of 50 Hz and 100 Hz, associated with a case study whereby workers presented with increased particulate in their nasal sinuses as a result of frequencies generated by nearby rotational machinery (Navarro et al. 2019; Pourmehran et al. 2023). Since then, researchers have analysed a variety of both acoustic and pulsating frequencies to determine the optimal conditions for drug delivery to the paranasal sinuses.

Maniscalco et al. (2006) reported that the deposition of drugs on the sinus walls could be increased by factors of 3 to 4.4 when nebulised aerosol flow was superimposed with pulsations at frequencies of 45, 120, and 200 Hz. Moeller et al. (2009) implemented pulsating flow *in vivo* at a frequency of 45 Hz and noted an increase in paranasal sinus deposition by 3–5%, along with a threefold enhancement in deposition within the nasal airway. Additionally, Farnoud et al. (2020) investigated the influence of different delivery angles ($45^{\circ }$ and $90^{\circ }$) and found that a $45^{\circ }$ angle combined with pulsating flow at 45 Hz resulted in a deposition increase in the maxillary sinus ranging from 0.22 to 0.25%.

Hosseini and Golshahi (2019) further examined the effects of a pulsating nebuliser in airway models across various age groups (2, 5, and 50 years), concluding that a frequency of 44.5 Hz enhanced maxillary sinus deposition. Furthermore, Pourmehran et al. (2020, 2021) explored the application of acoustic waves in nasal nebulisers, highlighting that a resonance acoustic frequency can improve deposition substantially. However, resonant frequencies are dependent on patient-specific anatomy, and consequently are difficult and time consuming to identify.

In the present study, we examine a circular pipe T-junction, which serves as a simplified representation of the maxillary ostium. While T-junctions are conventionally characterised by perpendicular connections, we hypothesise that modifying the radii of curvature ($R_{\text {c}}$) at the T-junction will affect flow penetration at the normal outlet. Additionally, we suggest that integrating this modified geometry with oscillatory flow may yield further improvements in performance. To evaluate these hypotheses, we investigate a spectrum of anterior and posterior $R_{\text {c}}$ at the T-junction, in conjunction with a pulsating inlet velocity condition at frequencies of 0, 30, 45, 60 and 75 Hz. A particle distribution of 1–70 $\upmu$m in diameter was used to represent common aerosolised particles used in respiratory drug delivery applications. The pulsating inlet is intended to model inhalation-only aerosol delivery used in nasal nebuliser therapy; exhalation is not part of the dosing phase and therefore is not simulated.

Insights obtained from the T-junction investigation are then translated to two patient-specific nasal cavity models, corresponding to postoperative and revision surgery states following sequential FESS procedures aimed at enlarging the maxillary ostium.

## Methodology

### Geometry models

To systematically assess the influence of local geometry, anterior and posterior $R_{\text {c}}$ were introduced in the simplified T-junction models as idealised design variables. These $R_{\text {c}}$ values do not represent direct anatomical measurements, which are impractical to define in irregular patient geometries, but rather serve as controlled analogues of surgical contouring (e.g. anterior wall resection or smoothing) that modify local flow redirection at the ostium.

**Fig. 1:**
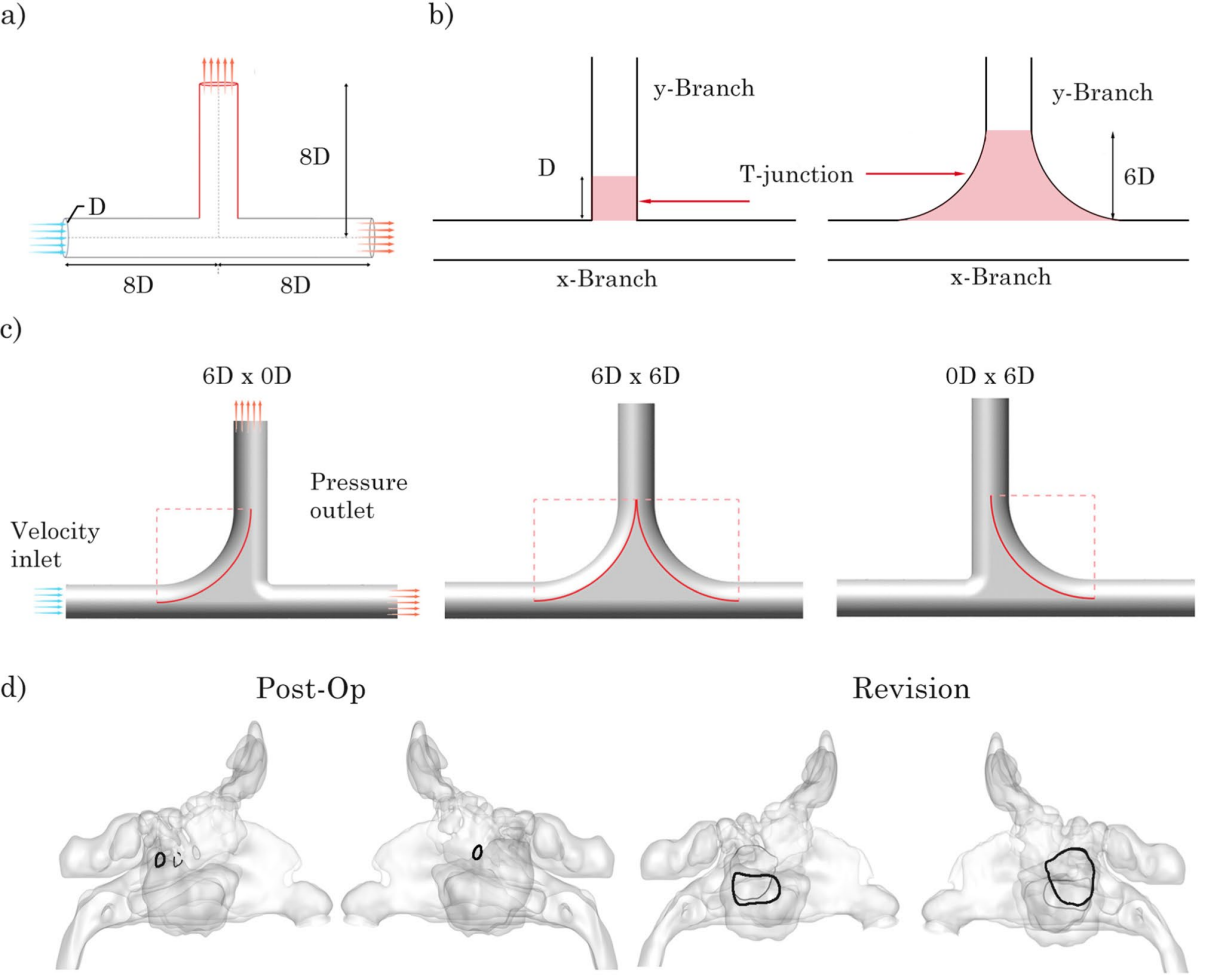
T-junction geometries used in this study. **a** Geometry layout and sizing. **b** Deposition locations *y*-branch, *x*-branch and T-Junction for 0*D* $\times$ 0*D* and 6*D* $\times$ 6*D* ($R_{\text {c}}$) models. $R_{\text {c}} = 6D$ was selected from prior parametric testing (Warfield-McAlpine et al. 2025) **c** Renderings of 6*D* $\times$ 0*D*, 6*D* $\times$ 6*D* and 0*D* $\times$ 6*D* ($R_{\text {c}}$) models. **d** Post-op and revision surgery models with maxillary ostium highlighted

The T-junction geometry was modelled in Ansys SpaceClaim (v2024R1) using a diameter of 3 mm. The entry, exit and normal branches were 8 D in length (Fig. 1). The $R_{\text {c}}$ on the anterior and posterior sides of the junction were varied, to parametrically assess particle deposition. Using $R_{\text {c}}$ of 0*D* and 6*D*, four models from combinations of the anterior and posterior alterations were produced. The 6*D* curvature was based on prior parametric testing (Warfield-McAlpine et al. 2025) showing this radius produced measurable changes in penetration without unrealistically enlarging the junction. The 8*D* entry/exit/normal branch lengths ensured sufficient distance for flow re-establishment downstream of the junction. These values therefore balance geometric sensitivity with anatomical plausibility.

**Fig. 2:**
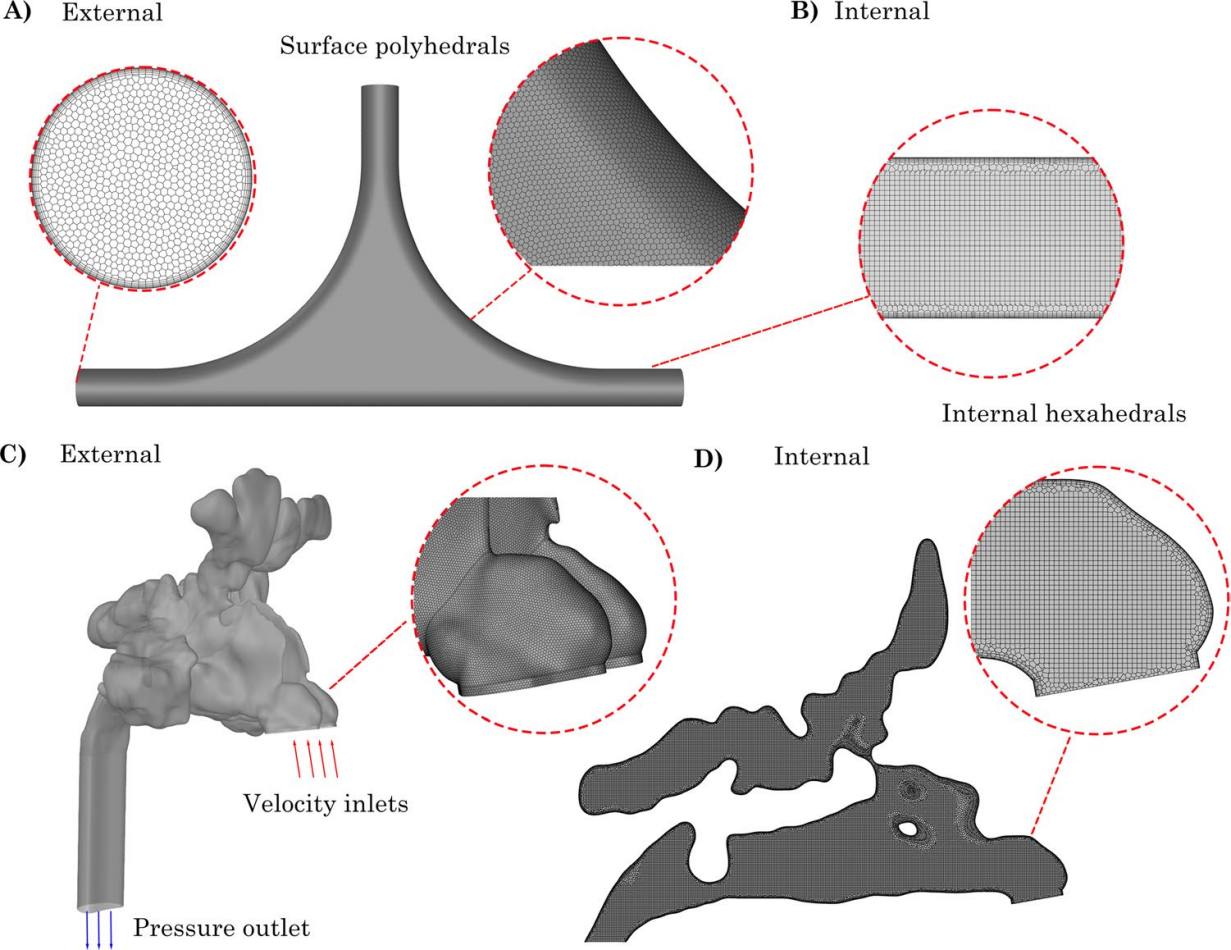
Poly-hexcore mesh applied to the 6*D* $\times$ 6*D* model. Right: External polyhedral cells. Left: Internal hexahedral cells

### Nasal cavity models

Patent specific nasal airway models were reconstructed from computed tomography (CT) scans of a 66-year-old female patient who had undergone two FESS procedures. These surgical interventions are designated as post-operative (Post-Op) and revision.

To define the velocity inlet boundary condition, the nasal vestibules of both models were sealed and extended by 1 mm to form a planar surface. Additionally, the nasopharynx was elongated to enhance numerical convergence.

### Transient and oscillatory flow settings

A fully developed laminar flow velocity profile was applied at the *x*-branch inlet to replicate physiological inhalation, where air enters through the nasal vestibule and travels axially before bifurcating into the maxillary ostium. In this model, the *y*-branch represents the ostium, which functions as a passage to the maxillary sinus. The profile was defined as:

1 $$\begin{aligned} u_{r} = 2u_{\text {avg}}\left( 1- \left( \frac{r}{R} \right) ^2 \right) \end{aligned}$$

where $u_{\text {avg}}$ is the average flow velocity, *r* is the radial coordinate from the pipe centre and *R* is the pipe radius.

This profile was then included in a sinusoidal function to induce pulsating flow under transient conditions using the following equation:

2 $$\begin{aligned} u(r,t) = u_{r}\left( 1 + \sin (2\pi f t ) \right) \end{aligned}$$

where $u_{r}$ is the laminar velocity profile given by Eq. (1), *f* is the frequency of oscillation, and *t* is the flow time in seconds. In the T-junction models, the oscillatory component at the *x*-branch inlet was imposed with a peak amplitude of 7.5 m/s (maximum inlet velocity 15 m/s). For the nasal airway simulations, a sinusoidal velocity boundary condition was imposed uniformly across the entire nostril inlet surface, with a peak amplitude of $\sim$ 2.5–2.8 m/s depending on nostril/model, consistent with our previous work (Warfield-McAlpine et al. 2025).

Four oscillating frequencies, *f* = 30, 45, 60 and 75 Hz, were investigated. To avoid startup effects, the simulation was first run for 0.0667 s to ensure full periods of all frequency conditions were completed. A time step of 1$\times 10 ^{-5}$ s was used for all cases which was associated with the mesh grid size to capture the laminar flow unsteadiness and maintain a $\textrm{CFL}$ (Courant–Friedrichs–Lewy) number of $\sim$ 1 for all frequencies. Table 1 summarises the unsteady simulation settings for the four frequencies and the associated Womersley numbers.

**Table 1:** Unsteady simulation settings and Womersley ($W_o$) number

Frequency (Hz)	Period (s)	Cycles startup	$W_o$
30	0.0333	2	10.8
45	0.0222	3	13.2
60	0.0167	4	15.2
75	0.0133	5	17.0

The Womersley number ($W_o$) is a dimensionless quantity that compares the pulsation frequency with the viscous effects within the flow simulation. It is expressed as follows:

3 $$\begin{aligned} W_o = D\sqrt{\frac{\omega \rho }{\mu }} \end{aligned}$$

where *D* is the diameter of the pipe, $\omega$ is the angular frequency of the oscillation, $\rho$ is the density, and $\upmu$ is the dynamic viscosity of the fluid. When $W_o$ exceeds one, the pulsating frequency becomes an important factor, and the flow can no longer be accurately predicted using a quasi-steady approximation.

Although the Reynolds number remains below the transitional threshold (Re $\le$ 1800), a Womersley number ($W_o$) of $\le$ 17 indicates that the flow is pulsatile and primarily governed by inertial forces, with viscous effects limited to the near-wall regions. Xu et al. (2017) examined pulsatile flow in smooth pipes and reported that, under similar conditions, the flow behaves in a quasi-laminar manner. As a result, all transient simulations in this study were conducted assuming laminar flow across all models. The flow regimes created by the pulsating frequency conditions are comprehensively analysed and documented in Warfield-McAlpine et al. (2025).

### Meshing

The geometry models were meshed using a poly-hexcore unstructured mesh in Ansys Fluent Meshing (v2024R1). The poly-hexcore mesh applies polyhedral cells to the surface boundaries and hexahedral cells to the bulk fluid region. A mesh independence study was performed using four mesh densities 0.4, 0.6, 1 and 1.3 million cells by exporting a velocity profiles at 1D, 2D and 3D from the T-junction on the *y*-branch on the control model (0D $\times$ 0D anterior and posterior $R_{\text {c}}$, respectively). A comparison of velocities indicated that independence was achieved with the 1 million cell mesh density. The associated mesh independence study is presented in Warfield-McAlpine et al. (2025), Table 2. The poly-hexcore mesh for the 6*D* $\times$ 6*D* model is shown in Fig. 2 (Table 2).

**Table 2:** Summary of mesh resolution parameters, where $N_{\text{cells}}$ is the number of mesh cells/elements, $\Delta$ is the internal core structured hex-mesh grid size, $N_{\text{pl}}$ is the number of prism layers, and $h_{\text{pl}}$ is the first prism layer height

Mesh	$N_{\text{cells}}\times 10^6$	$\Delta$ (mm)	$N_{\text{pl}}$	$h_{\text{pl}}$ (mm)
Mesh 1	0.40	0.150	6	0.006
Mesh 2	0.60	0.145	6	0.006
Mesh 3	1.00	0.100	6	0.006
Mesh 4	1.30	0.080	6	0.006

**Fig. 3:**
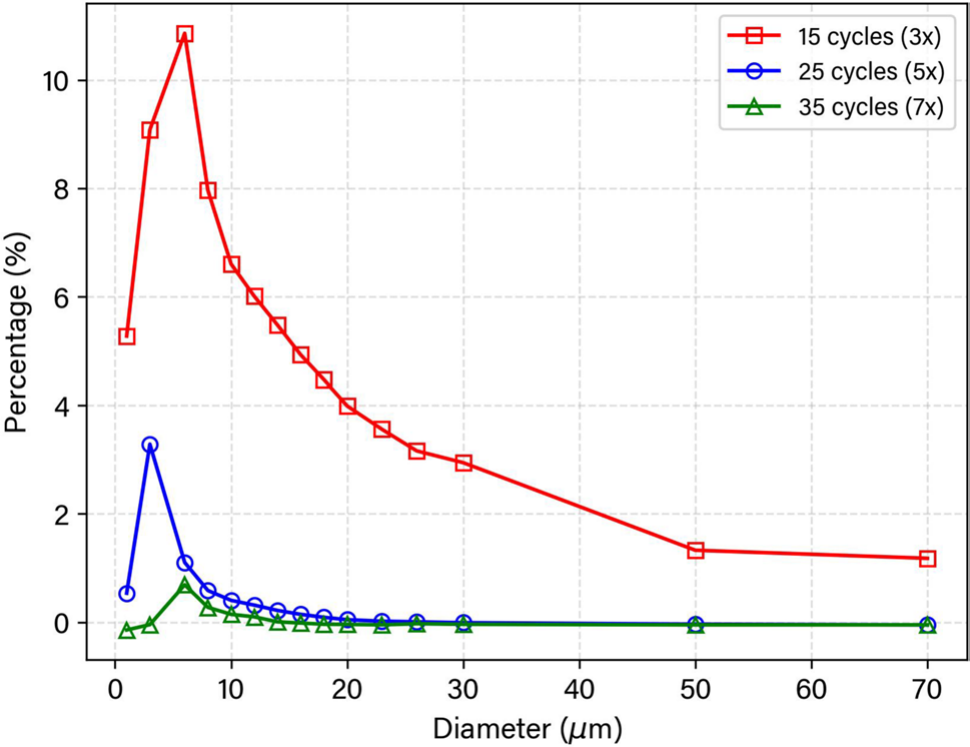
Percentage of incomplete particles following 3$\times$, 5$\times$, & 7$\times$, tested at 75 Hz representing 15, 25, and 35 additional cycles, respectively

**Fig. 4:**
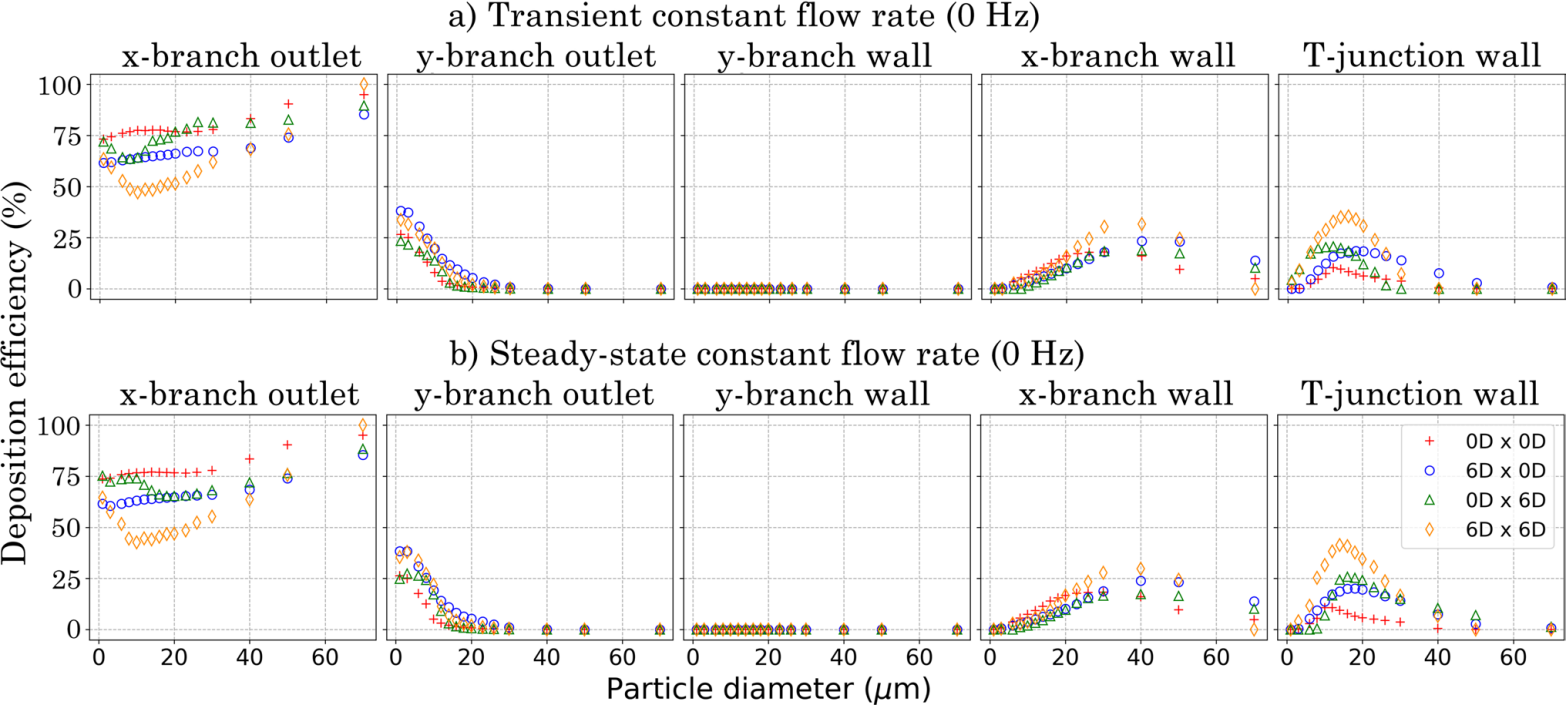
Particle mass fractions in local regions for all particles (1–70 $\mu$m) for models 0*D* $\times$ 0*D* ( ); 6*D* $\times$ 0*D* ( ); 0*D* $\times$ 6*D* ( ); and 6*D* $\times$ 6*D* ( )

Due to the complexity of nasal geometries, a fault-tolerant workflow in Ansys Fluent (v2024R1) was used to wrap surfaces and prevent leakage caused by CT segmentation. Element sizing strategies included applying a 0.2-mm body of influence for internal cells, curvature refinement on surfaces and edges, and proximity refinement across all surfaces, with element sizes ranging from 0.4 to 8 mm. Six prism layers were added at the fluid boundary using the last-ratio method, a first cell height of 0.004 mm and a transition ratio of 0.26. Additionally, two peel layers were incorporated to ensure a smooth transition between polyhedral and hexahedral elements. The resulting mesh contained approximately 5 million elements. Mesh independence was confirmed following the approach outlined in Inthavong et al. (2018). These models have been previously published in Siu et al. (2020, 2021) and Warfield-McAlpine et al. (2025).

### CFD modelling

Transient simulations were performed under a laminar flow regime using Ansys Fluent (v2024R2). Spatial discretisation for gradient, pressure and momentum was applied using the least squares cell-based, second order and second-order upwind schemes, respectively. The SIMPLE algorithm was used for the pressure–velocity coupling. The simulations were deemed converged when the local residual error fell below 1 $\times 10^{-5}$ for continuity and momentum. Oscillatory frequencies of 0, 30, 45, 60 and 75 Hz were applied to the inlet boundary to generate pulsating flow using Eq. (2). Further details on the flow characteristics and continuous phase methodology are provided in our previous work (Warfield-McAlpine et al. 2025).

### Discrete phase

Discrete phase modelling was performed using a ‘one-way coupled’ Euler–Lagrangian model in Ansys Fluent (v2024R2). This model approximates the trajectories of individual particles by integrating the force balance on the particle. The force balance includes the particle inertia and forces acting on the particle and is shown in Eq. (4),

4 $$\begin{aligned} m_p\frac{{\text {d}} \varvec{u}_{i}^{p}}{{\text {d}}t} = \varvec{f}_{\text {d}} + m_p\varvec{g} \end{aligned}$$

where superscript *p* denotes the particle phase and $\varvec{f}_d$ is the drag force given by Eq. 5.

5 $$\begin{aligned} \varvec{f}_D = m_p \frac{( \varvec{u}_{i}^g - \varvec{u}_{i}^p)}{\tau _{\text {p}}} \end{aligned}$$

where *g* and *p* refer to the fluid and particle phases, respectively, and $\tau _{\text {p}}$ is the particle response time given by:

6 $$\begin{aligned} \tau ^p = \frac{d_{{\text {p}}}^{2} \rho ^{p} C_{{\text {c}}}}{18 \mu } \frac{24}{C_{\text {d}} \textrm{Re}} \end{aligned}$$

where $C_{\text {d}}$ is the drag coefficient and $C_{\text {c}}$ is the Cunningham correction factor which is used to adjust the Stokes drag law to represent sub-micron particles and is considered negligible for this study.

A particle diameter ($d_p$ distribution range of 1–70 $\mu$m) was simulated to represent drug delivery applications. Following an initial simulation time of 0.0667 s, to establish the flow field, the flow time was reset and 150 particle streams were injected per time step for 6667 time steps at a time step size of 1 $\times 10^{-5}$ s (every time step from 0 to 0.0667 s). This gave a total of 1 million particle streams modelled for each $d_{\text {p}}$. An initial particle velocity of 4.38 m/s (associate with a maximum *Re* = 1800, under pulsating conditions) was imposed and the particle starting points were randomised at the inlet face. A test for determining a sufficient time for particle clearance was evaluated to reduce the number of incomplete particles that may persist within the domain, where the total time is given in multiples of the injection cycles at 75 Hz and is presented in Fig. 3. Particle integration tracking was performed under the Runge–Kutta scheme to ensure accurate modelling of particles < 5 $\upmu$m following findings from previous work (Warfield-McAlpine et al. 2024). Following the analysis, the simulation was then run for a remaining 0.33 s to ensure particle clearance. Particle injection details are provided in Table 3.

**Fig. 5:**
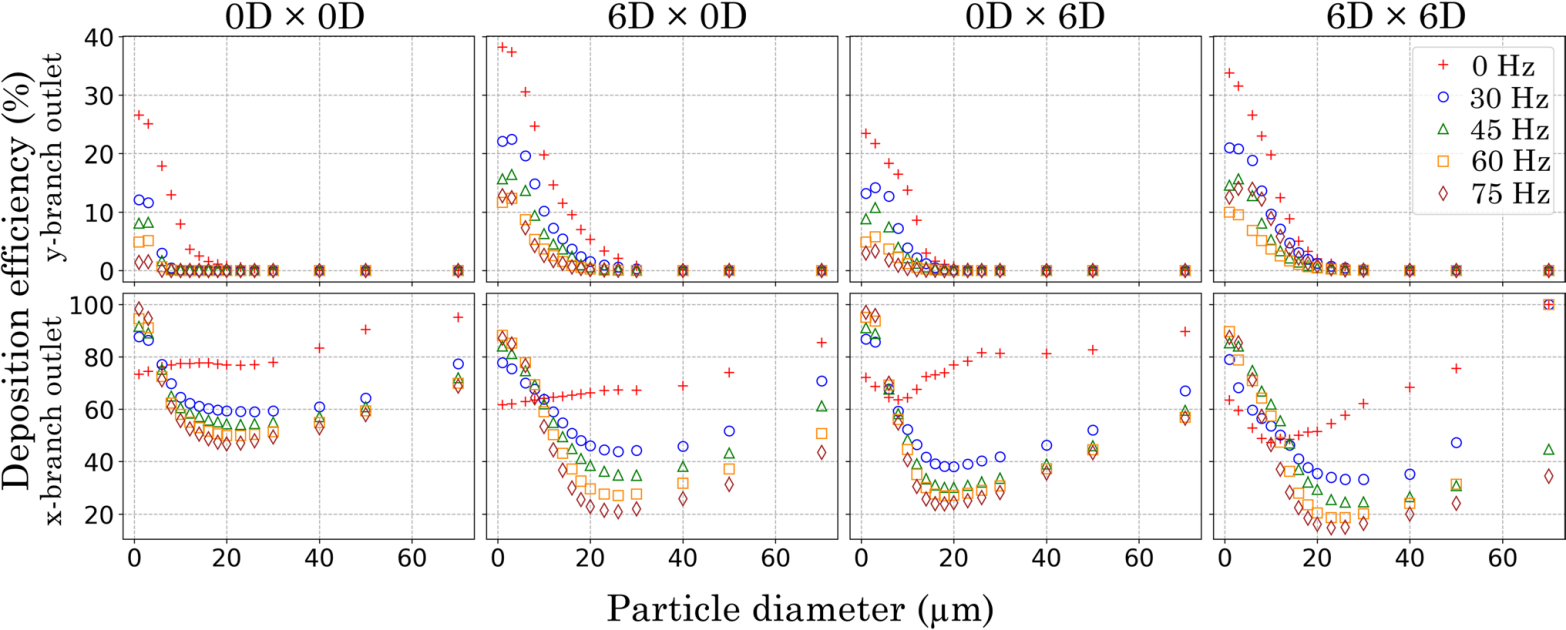
Particle mass fraction at geometry for a particle distribution of 1–70 $\upmu$m for models 0*D* $\times$ 0*D*, 18 mm $\times$ 0*D*, 0*D* $\times$ 6*D* and 6*D* $\times$ 6*D*

**Table 3:** Summary of particle injection periods for T-junction and Nasal airway models

*f* (Hz)	Injection-time (s)	Injection-cycles	Clearance-cycles
0	0.0667	N/A	N/A
30	0.0667	2	10
45	0.0667	3	15
60	0.0667	4	20
75	0.0667	5	25

*f* is the pulsating frequency, Injection-time is the period of injection, Injection-cycles is the number of oscillatory cycles that occur during the injection time. Clearance-cycles is the number of additional cycles during the clearance period associated with 5$\times$ the injection time

Mass fractions were recorded at the wall surfaces and the exits through the *x*- and *y*-branch outlets using Eq. (7). The *x*-branch label represents the wall surface region along the axial flow direction and is confined by the diameter of the tube. As the T-junction geometry curvature varies considerably between the 0*D* and 6*D* $R_{\text {c}}$ models, the T-junction wall surface was represented as the region between 1 D and 2 D from the base of the model for the 0*D* $\times$ 0*D* model and between 1*D* and 6*D* for the 6*D* $R_{\text {c}}$ models (see Fig. 1).

7 $$\begin{aligned} \mathrm {mass\;fraction}\;(m_f) = \frac{\Delta m_{\textrm{zone}}}{\Delta m_{{\mathrm zone}} + \Delta m_{\mathrm {remaining\;zones}}} \end{aligned}$$

Particle injections within the nasal geometries were distributed evenly between the two nostrils, with initial velocities calibrated to reflect the average flow velocity corresponding to a flow rate of 7.5 L/min per nostril. Nasal boundary surfaces and nostril inlets were set to trap and reflect, respectively. The nasopharynx was defined as an outflow escape boundary, ensuring that particles exiting the domain did not re-enter. Although the inlet profile included a sinusoidal pulsation (Eq. 2), the velocity oscillated between zero and $2u_r$ without reversing, such that no backflow occurred at the outlet; instead, transient pressure gradients within the T-junction governed particle redirection towards the ostium during deceleration phases.

As particle deposition in the nasal cavity is primarily governed by inertial impaction, the inertial parameter (IP) was used to normalise the deposition behaviour across the different $d_\text {p}$s (Kelly et al. 2004; Shi et al. 2008; Inthavong et al. 2011; Warfield-McAlpine et al. 2024) given by:

8 $$\begin{aligned} \text {IP} = d_\text {a}^2 Q \end{aligned}$$

where $d_\text {a}$ is the aerodynamic diameter (in ${\upmu }$m) and *Q* is the flow rate (in $\hbox {cm}^3$/s).

## Results

### Steady-state particle mass fraction in branches

Figure 4 compares steady state and pulsatile flow deposition efficiency at 0 Hz for all T-junction models. At 0 Hz, the sinusoidal function has zero amplitude, resulting in a constant flow regime Re = 900; therefore, the steady-state solution was computed using the corresponding flow field.

Models 0*D* $\times$ 0*D* and 6*D* $\times$ 0*D* showed negligible deposition efficiency variation at all locations in the geometry. However, models 0*D* $\times$ 6*D* and 6*D* $\times$ 6*D* exhibited significant variances at both the *x*- and *y*-branch outlets and the T-junction wall. This difference was attributed to unsteady laminar mixing at these locations and the recirculation of smaller particles, which was absent in the steady-state simulation. Furthermore, the transient simulation showed a 6% increase in incomplete particles for the particle range of 1-8 $\upmu$m. Although these inconsistencies were present in the steady-state solutions, the magnitude of these differences were minimal. Therefore, the results from the transient simulation at 0 Hz were used as a control case and are discussed in comparison with the pulsating flow at 30, 45, 60 and 75 Hz.

**Fig. 6:**
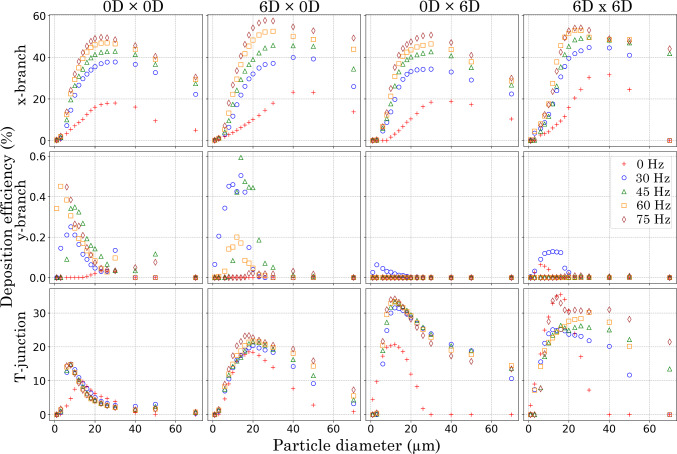
Particle mass fraction at geometry for a particle distribution of 1–70 $\upmu$m for models 0*D* $\times$ 0*D*, 18 mm $\times$ 0*D*, 0*D* $\times$ 6*D* and 6*D* $\times$ 6*D*

**Fig. 7:**
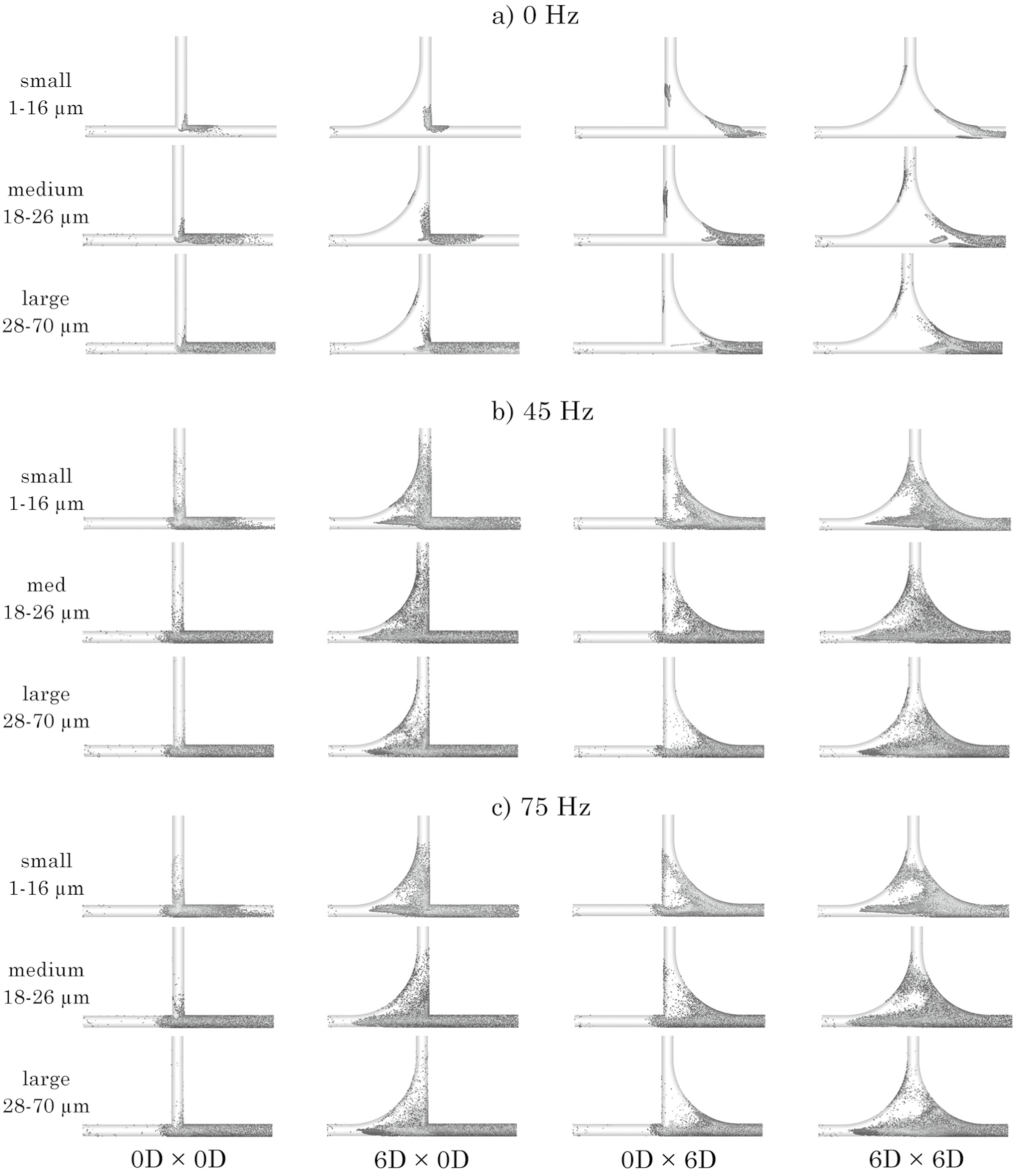
Particle deposition patterns on boundary walls from the *x*-*y* plane (side view) at 0, 45 and 75 Hz for particle ranges 1–16 $\upmu$m, 18–26 $\upmu$m and 28–70 $\upmu$m

### Particles exiting the flow

The particle mass flux at outlets in each geometry for all oscillating flows is presented in Fig. 5. At all frequencies (0, 30, 45, 60 and 75 Hz), the *x*-branch outlet shows a relatively high exiting mass flux for smaller $d_\text {p}$s (< 20 $\upmu$m), although at 0 Hz, there is a significant decrease beyond 20 $\upmu$m, and with a peak for 70 $\upmu$m particles exiting for model 6*D* $\times$ 6*D*. However, this model had the least particle mass leaving the *x*-branch outlet for the remaining particles diameters (1–50 $\upmu$m).

In the 0*D* $\times$ 0*D* model at 0 Hz, approximately 80% of particles ranging from 0 30 $\upmu$m exited through the *x*-branch outlet, with the proportion of particles exiting increasing as the $d_\text {p}$ exceeded 30 $\upmu$m. Under a pulsating boundary condition, an increase in smaller particles (< 6 $\upmu$m) depositing was observed. As the pulsating frequency was raised from 30 to 75 Hz the percentage of smaller particles (<10 $\upmu$m) at the *x*-branch outlet increased in all models. In contrast, the mass flux of particles with diameters >10 $\upmu$m that exited the domain decreased (Fig. 5).

The *y*-branch sits perpendicular to the flow, and this makes it difficult for larger-diameter particles to penetrate the *y*-branch and escape through its outlet. There is a distinct trend where the exiting mass flux decreases sharply with increasing $d_\text {p}$. At 0 Hz, particles exiting the *y*-branch outlet were limited to diameters below 30 $\upmu$m. With the addition of pulsating frequencies (30, 45, 60 and 75 Hz), the particle sizes exiting the *y*-branch outlet were further reduced to below 20 $\upmu$m in models that included an anterior $R_{\text {c}}$. Although the pulsating frequency increased flow unsteadiness, it resulted in fewer particles leaving the *y*-branch outlet. Higher pulsation frequencies generated vortical structures at the junction that trapped particles near the walls and redirected them towards the axial branch. This reduced net transport through the *y*-branch outlet, with particles instead depositing on the geometry walls or exiting axially. These flow features were consistent with the Q-criterion iso-surfaces shown in the supplementary material (Fig. S1), where increasing frequency produced stronger and more compact vortices that remained confined near the junction, thereby limiting side-branch penetration despite greater overall unsteadiness.

The addition of a $R_{\text {c}}$ at either the anterior or posterior side of the T-junction led to a reduction in particles entering the *y*-branch and subsequently leaving through its outlet. The control model (0*D* $\times$ 0*D*) and the posterior $R_{\text {c}}$ model (0*D* $\times$ 6*D*) exhibited the lowest mass fraction of particles exiting the *y*-branch outlet at all pulsating frequencies. In contrast, the anterior $R_{\text {c}}$ models (6*D* $\times$ 0*D* and 6*D* $\times$ 6*D*) showed the highest mass fraction of particles leaving the *y*-branch outlet for all particle sizes, with a peak mass fraction of 38% observed for the smallest particle size of 1 $\upmu$m.

### Particle deposition on the walls

Figure 6 shows particle deposition efficiency on the T-junction walls. At 0 Hz, particle deposition on the *x*-branch wall was negligible across all models. However, the introduction of pulsatile flow led to an increase in deposition on the *x*-branch wall, which intensified with higher pulsation frequencies. Under the pulsating conditions, particle deposition increased with particle size, peaking at approximately 40–50% and decreasing as $d_\text {p}$ further increased.

At all pulsating frequencies (30, 45, 60 and 75 Hz), anterior $R_{\text {c}}$ models (6*D* $\times$ 0*D* and 6*D* $\times$ 6*D*) exhibited the highest particle deposition for all $d_p$s (1 70 $\mu$m) at the *x*-branch wall.

On the *y*-branch wall (normal to flow direction), low deposition efficiency was recorded under all pulsating frequency and $R_{\text {c}}$ conditions. Deposition efficiency of small particles (1–20 $\mu$m) improved with either no $R_{\text {c}}$ (0*D* $\times$ 0*D*) or anterior $R_{\text {c}}$ only (6*D* $\times$ 0*D*). Pulsating frequency had a significant effect on deposition efficiency for these models, with improved deposition linked to increasing frequency in the 0*D* $\times$ 0*D* model and frequencies of 30 and 45 Hz in the 6*D* $\times$ 0*D* model. Minimal deposition was observed for particle sizes > 20 $\mu$m. For posterior $R_{\text {c}}$ models (0*D* $\times$ 6*D* and 6*D* $\times$ 6*D*) deposition on the *y*-branch wall was only observed at a pulsating frequency of 30 Hz.

Deposition on the T-junction wall showed significant differences among the models. This variation was expected due to differences in the surface boundary allocation (see Fig. 1b) and the associated surface area. At 0 Hz, the 6*D* $\times$ 6*D* model exhibited the largest deposition for particle sizes ranging from 1–24 $\upmu$m, while the anterior $R_{\text {c}}$ model showed the highest deposition for particles sized 24–70 $\upmu$m.

**Fig. 8:**
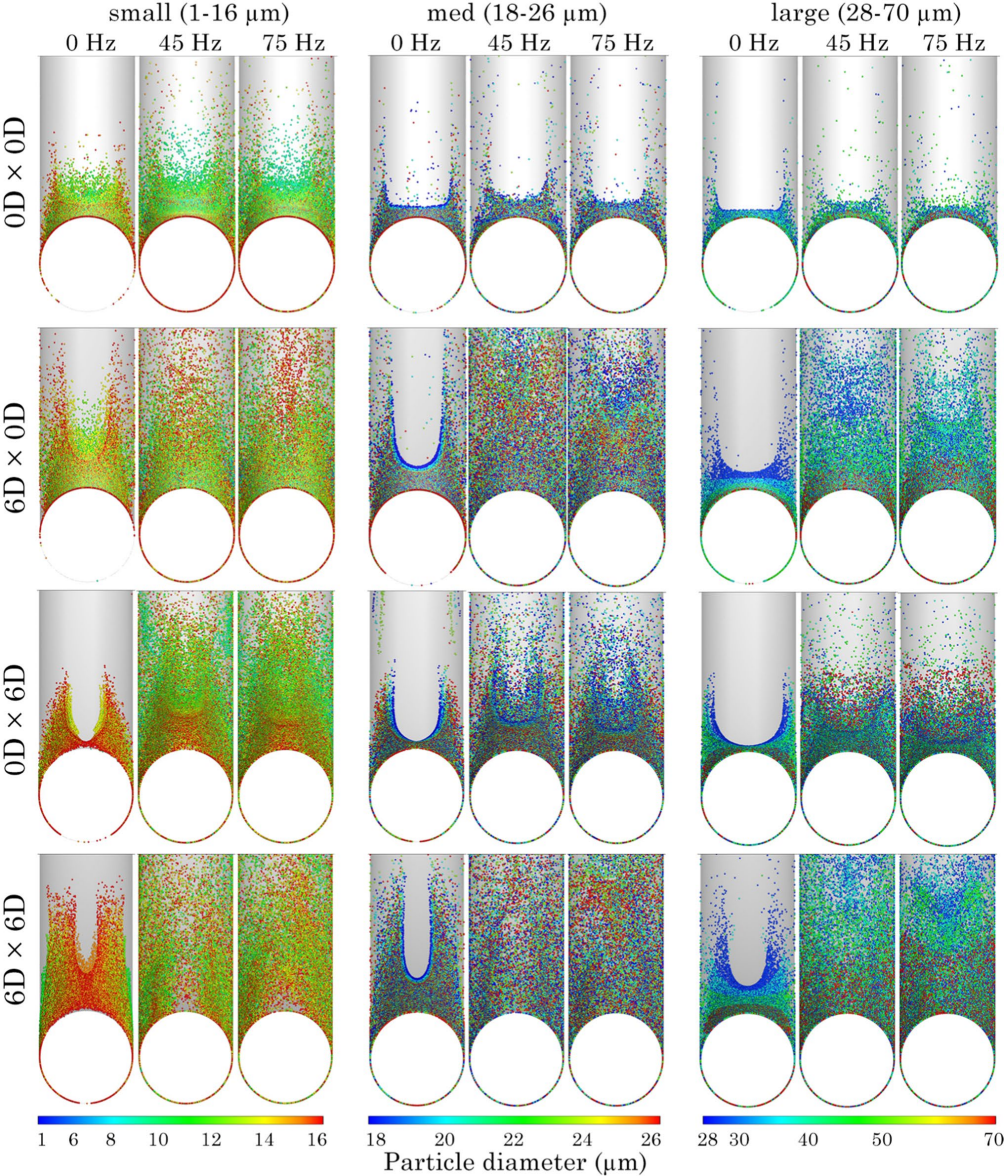
Particle deposition patterns on boundary walls in the axial direction for 0, 45 and 75 Hz. Showing particle size distribution at the *y*-branch and T-junction walls, for particle ranges 1–16 $\upmu$m, 18–26 $\upmu$m and 28–70 $\upmu$m

Particle deposition on the boundary walls are shown in the side view (*x*–*y* plane), in Fig. 7, and the axial view (*y*-*z* plane) in Fig. 8 to provide the visual understanding of the fluid-particle behaviour. From the side view, the majority of particles deposit on the exit length of the *x*-branch for cases with no anterior $R_{\text {c}}$. With an anterior $R_{\text {c}}$, particle deposition is found earlier in the *x*-branch and at a greater quantity in the *y*-branch. At 0 Hz, particle deposition is primarily in the exit region of the *x*-branch and at small localised points in the *y*-branch. At a frequency of 45 Hz, particle deposition is increased in all regions of the geometry, notably at the *y*-branch wall. Although increasing the pulsating frequency to 75 Hz led to additional velocity fluctuations in the flow field, particle deposition in the exit end of the *y*-branch wall was reduced.

In general, a 0 Hz flow (no flow oscillation) showed particles readily exited or deposited on the exit sections of the tubes, while introducing a frequency of 45 or 75 Hz provides the ability for particles to move into the *y*-branch through the oscillating flow. Additionally, the presence of an anterior $R_{\text {c}}$ further enhanced the penetration into the *y*-branch. There was a wider deposition distribution pattern of medium particles (18–26 $\upmu$m), especially for regions of curvature, $R_{\text {c}}$. The smaller particles (1–16$~\upmu$m) demonstrated greater deposition distribution in the *y*-branch, while the larger particles (26–70 $\mu$m) remained more aligned with the *x*-branch.

Figure 8 presents particle deposition by size range at the *y*-branch and T-junction walls from an axial view of the geometry. In the control model (0*D* $\times$ 0*D*), particle deposition was limited to diameters < 40 $\upmu$m, primarily within the lower end of the *y*-branch. This trend remained consistent across all frequency conditions. Introducing an anterior $R_{\text {c}}$ increased both the magnitude and height of deposition, with significantly higher deposition observed at pulsation frequencies of 45 and 75 Hz, though the particle size distribution remained constant.

The posterior $R_{\text {c}}$ model (0*D* $\times$ 6*D*) displayed a similar trend in deposition pattern but with a greater amount of larger particles depositing along the T-junction wall. In the combined anterior and posterior $R_{\text {c}}$ model (6*D* $\times$ 6*D*), deposition increased throughout the T-junction geometry, particularly for the large particle size range (1–70 $\upmu$m).

### Radial particle positions in the control model

**Fig. 9:**
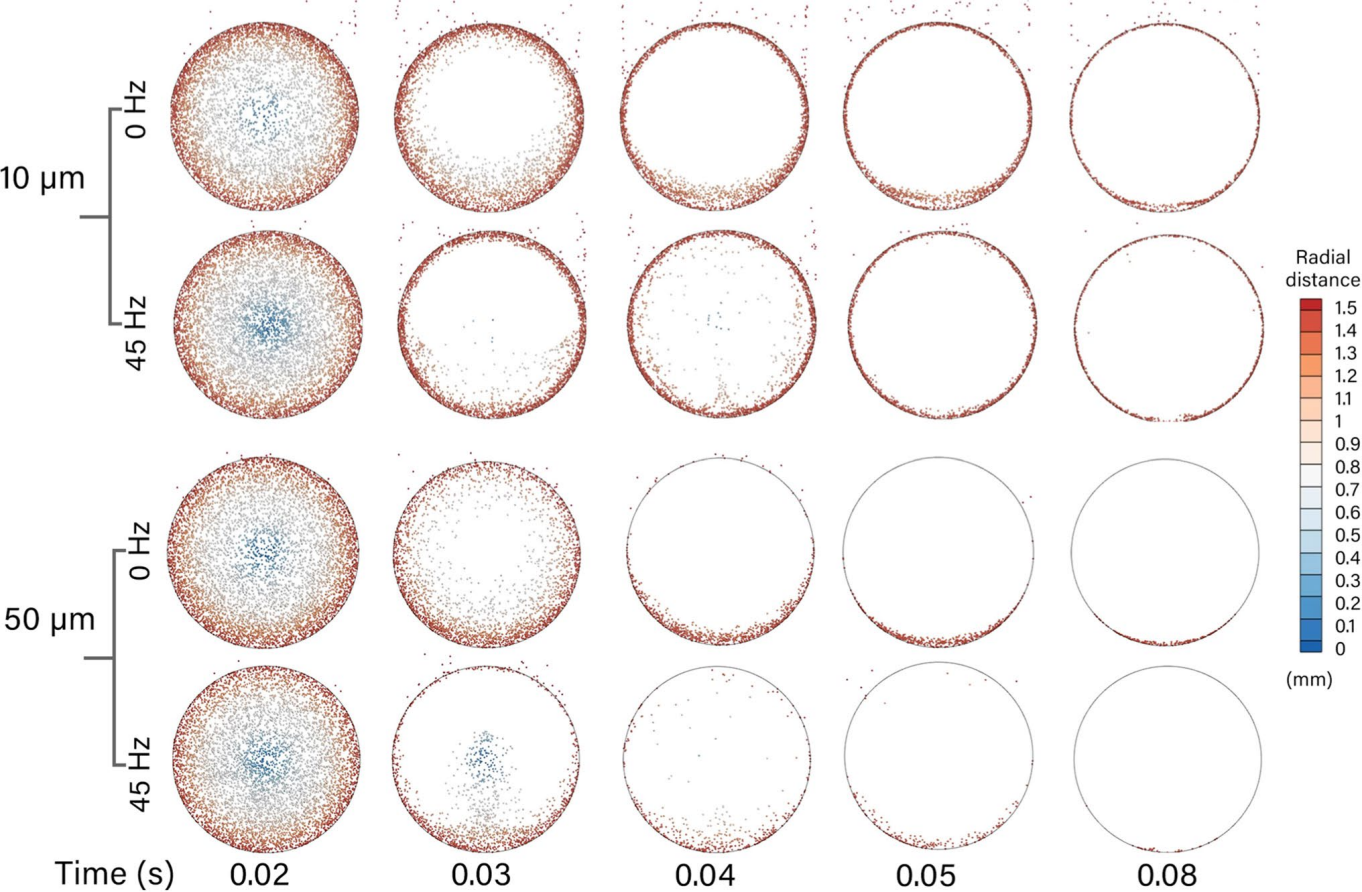
Radial distance travelled by particle sizes 10 $\upmu$m and 50 $\upmu$m in the 0*D* $\times$ 0*D* model at 0 and 45 Hz. (Multimedia full complete cycles available online)

The particle radial positions in the *x*-branch was recorded in the control model (0*D* $\times$ 0*D*) at 0 and 45 Hz, see Fig. 9. The injection time was reduced to 0.022 s, representing one full period at 45 Hz, and particle positions were recorded every 4 time steps to provide enhanced visualisation. The 10 $\upmu$m particle at 0 Hz showed particles moving through the tube and the remaining particles are caught in the low velocity regions near the walls. There are more in the lower *x*-branch since these accumulate over the entire tube length, while the upper region is influenced by particles entering the *y*-branch. At 45 Hz, there were fewer residual particles in the *x*-branch, as the oscillating enhanced penetration into the *y*-branch. For the larger particles of 50 $\upmu$m, there was a similar behaviour, where nearly all the particles cleared the *x*-branch as fewer residual particles were found in the domain.

### Aerosol deposition efficiency in the nasal airway

**Fig. 10:**
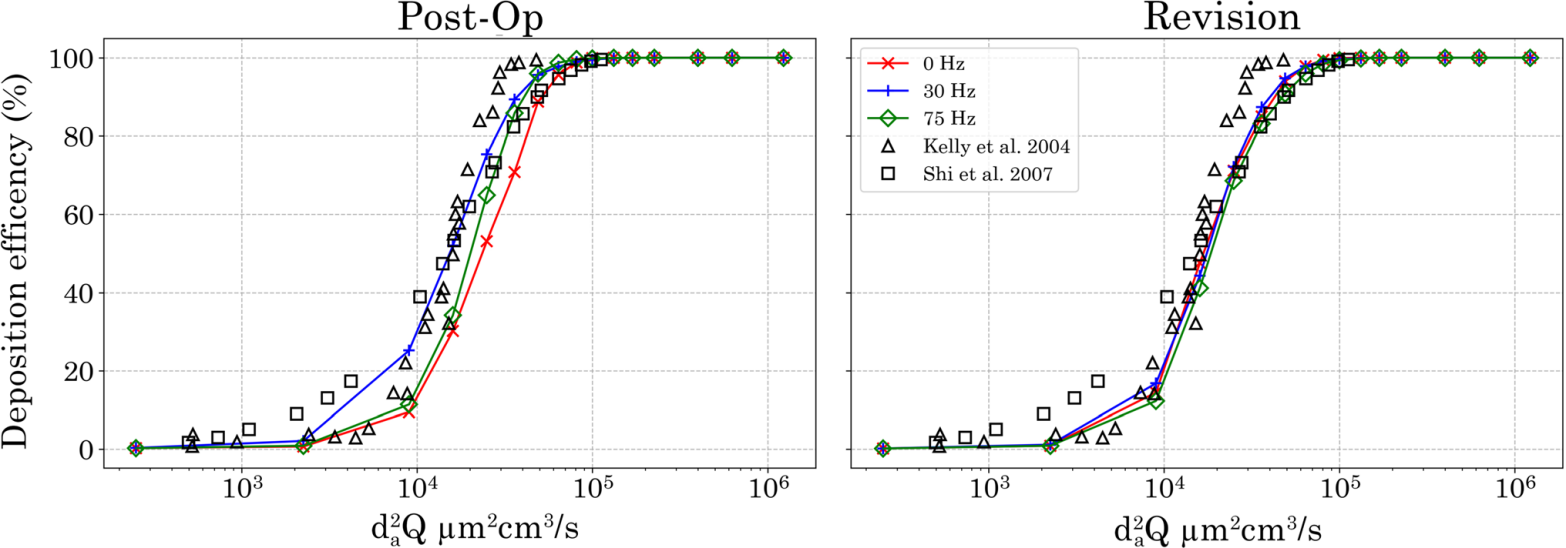
Comparisons of the inertial particle deposition simulation using pulsating flow conditions of 0 Hz, 30 Hz, and 75 Hz against experimental (Kelly et al. 2004) and numerical (Shi et al. 2008) data

The deposition efficiency was evaluated for the post-op and revision models. Figure 10 shows the deposition efficiency for both post-op and revision models aligns closely to experimental (Kelly et al. 2004) and numerical data (Shi et al. 2008) under pulsating frequency conditions 0, 30 and 75 Hz.

The post-op model was observed to be sensitive to the applied pulsating frequency with variances observed of 0, 30 and 75 Hz boundary conditions. The 30 Hz boundary condition generated the highest deposition efficiency and aligned best to experimental data for all particle sizes, while the 75 Hz boundary condition had minimal variance when compared with the 0 Hz boundary condition.

In contrast, the revision surgery model demonstrated lower sensitivity to pulsatile frequency, with all conditions aligning well with experimental and numerical results. Although deposition efficiency remained comparable across frequencies, a slight improvement was observed at 30 Hz.

### Particle distribution in the nasal airway

**Fig. 11:**
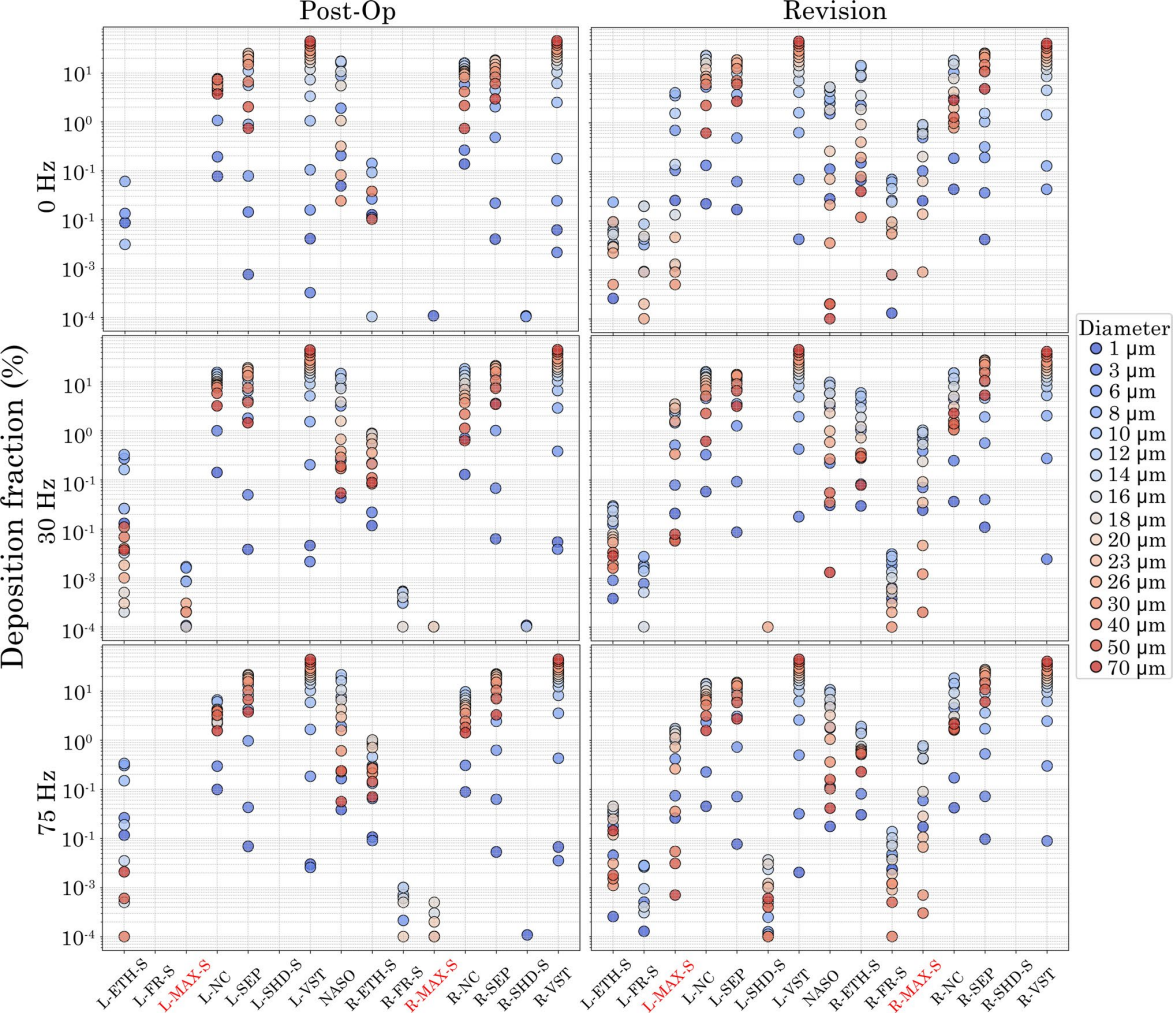
Localised deposition efficiency in the post-op and revision nasal airways under pulsating flow conditions.(L: Left, R: Right, S: Sinus, ETH: Ethmoid, FR: Frontal, MAX: Maxillary, NC: Nasal cavity, SEP: Septum, VST: Vestibule, NASO: Nasopharynx)

Figure 11 illustrates the influence of pulsation frequency and particle size on regional deposition fractions across post-op and revision nasal cavity models. In the post-op model, deposition was highly sensitive to frequency, with the 30 Hz condition generating higher deposition fractions across most anatomical regions and particle sizes compared to both 0 Hz and 75 Hz. Conversely, the 75 Hz condition exhibited deposition patterns comparable to the 0 Hz, indicating minimal added benefit at higher frequencies.

Conversely, the revision model displayed relatively uniform deposition across all frequencies, indicating reduced sensitivity to pulsatile effects. Regardless of frequency, particle size significantly influenced deposition: Larger particles ($\ge$ 20 $\upmu$m) showed substantially higher deposition fractions, particularly in anterior regions such as the nasal vestibules (L-VEST, R-VEST) and axial flow regions such as the nasal septum and cavities (L-SEP, R-SEP, L-NC, R-NC), while smaller particles ( < 10 $\upmu$m) demonstrated minimal deposition. These results underscore the dominant role of inertial impaction for larger particles and highlight how surgical geometry and pulsatile airflow interact to influence deposition patterns.

**Fig. 12:**
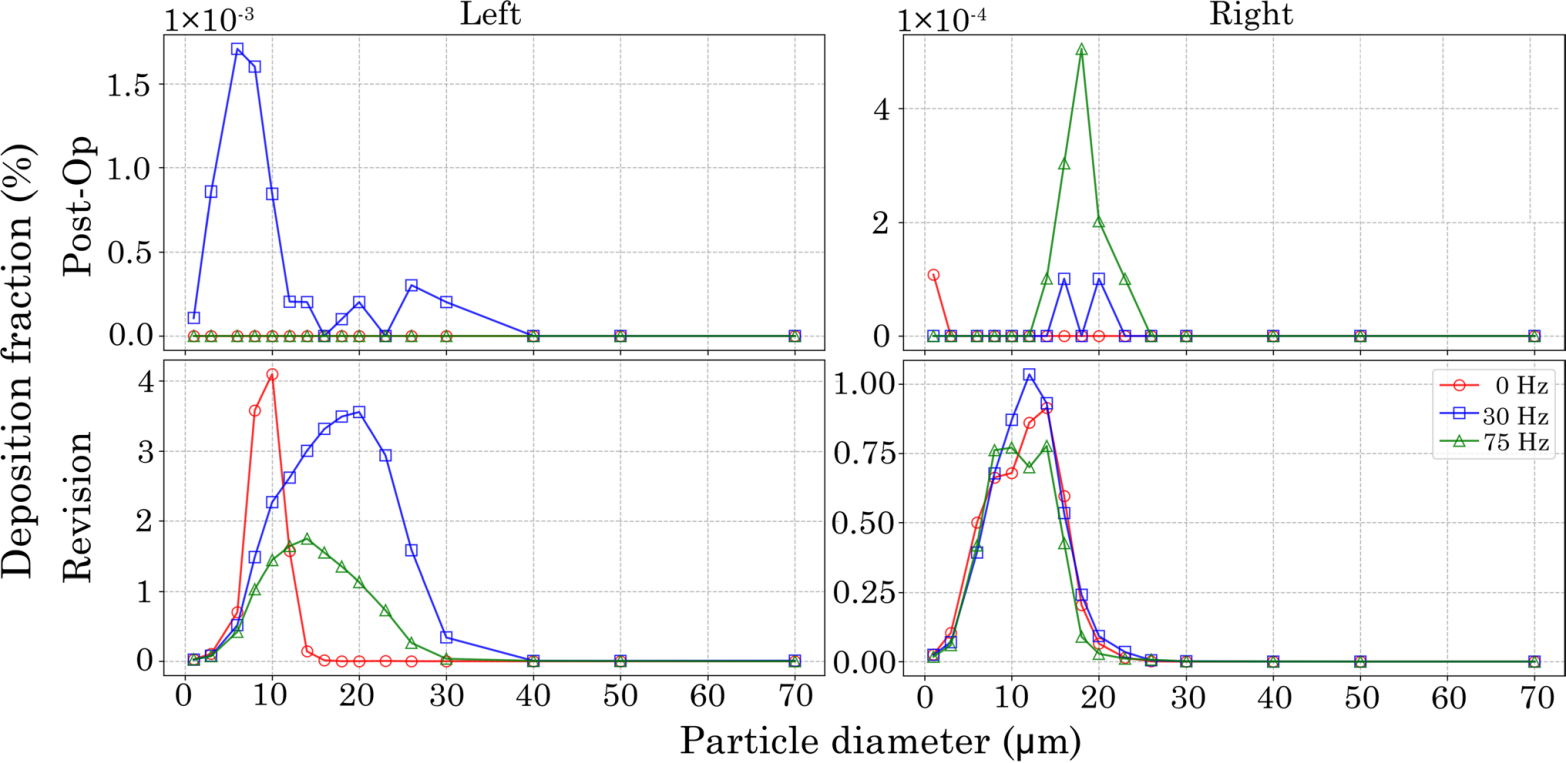
Localised particle deposition fraction in the right and left maxillary sinus for post-op and revision model

Figure 12 shows localised particle deposition within the left and right maxillary sinuses for both post-op and revision models. In the post-op model, deposition in the left maxillary sinus occurs exclusively under the 30 Hz pulsatile condition and is limited to particles < 40 $\upmu$m. Conversely, the right maxillary sinus exhibits enhanced deposition under the 75 Hz condition, particularly for particles ranging from 12–28 $\upmu$m.

In the revision model, both the left and right maxillary sinuses exhibit substantially increased particle deposition, attributed to the extensive surgical intervention that significantly enlarged the maxillary ostia. The size distribution of deposited particles is influenced by the applied pulsation frequency. At 0 Hz, deposition is restricted to particles within the 3–16 $\upmu$m range. Increasing the frequency to 30 Hz broadens the deposition range to 3–40 $\upmu$m. While a similar distribution is seen at 75 Hz, the overall deposition fraction is approximately halved. Notably, deposition at the right maxillary ostium appears largely unaffected by pulsation frequency, exhibiting a consistent size distribution and magnitude across all conditions.

### Deposition patterns in the nasal airway

**Fig. 13:**
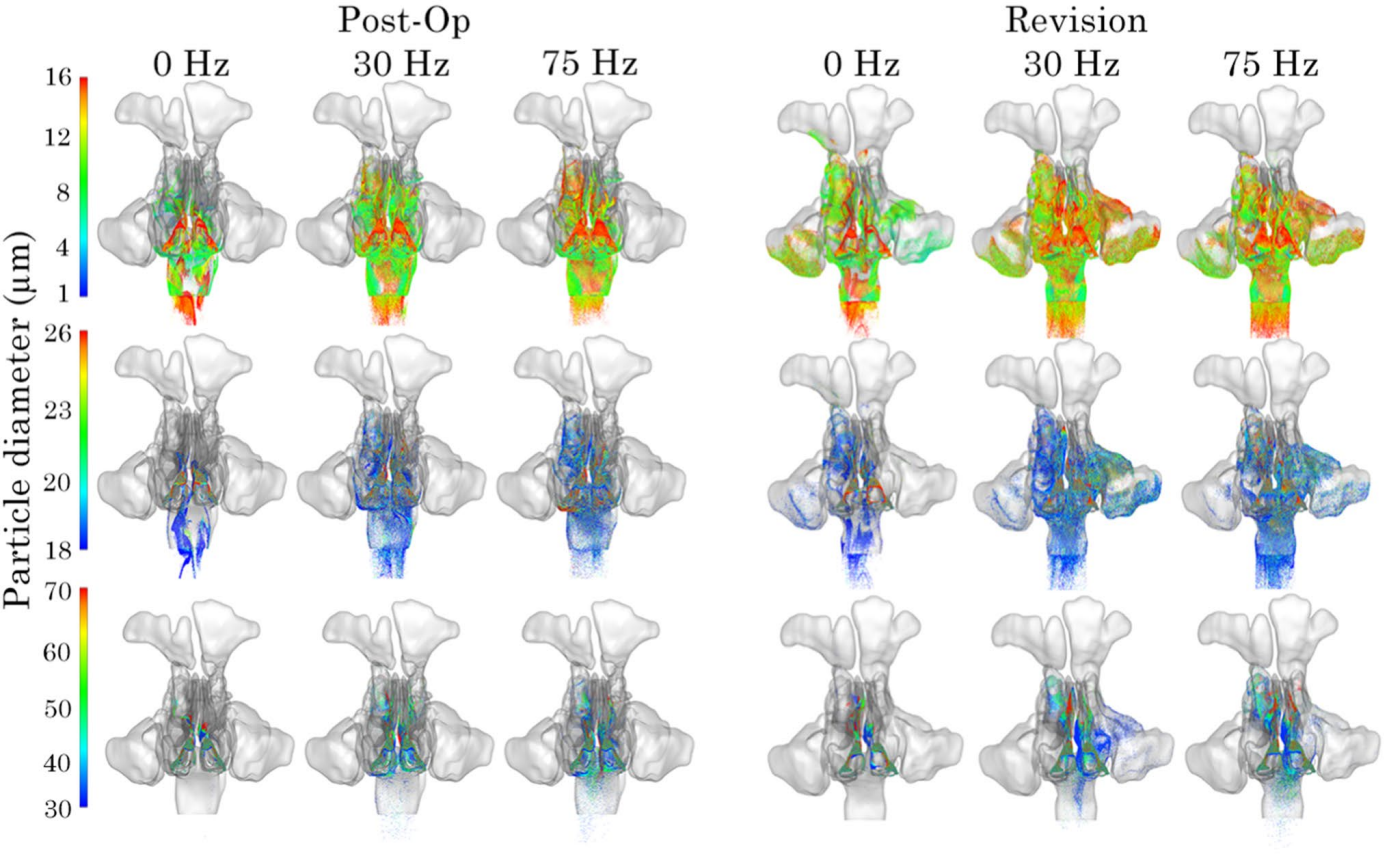
Frontal view of particle deposition patterns in the nasal airway 0, 45, and 75 Hz. Showing particle size distribution for particle ranges 1–16 $\upmu$m, 18–26 $\upmu$m and 28–70 $\upmu$m

**Fig. 14:**
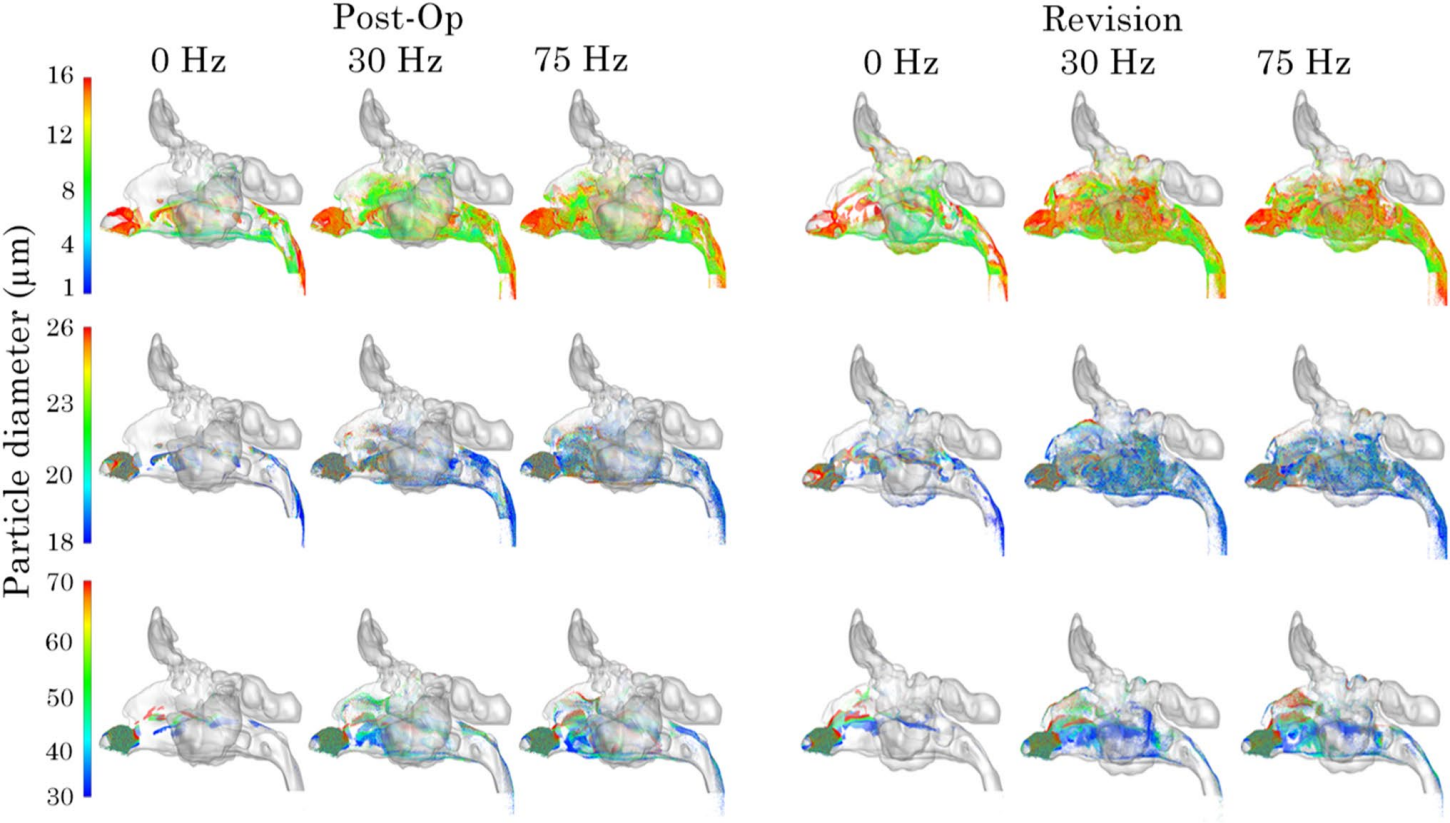
Side view of particle deposition patterns in the nasal airway 0, 45 and 75 Hz. Showing particle size distribution for particle ranges 1–16 $\upmu$m, 18–26 $\upmu$m and 28–70 $\upmu$m

The deposed particle distribution for the post-op and revision models at pulsating frequencies of 0, 30 and 75 Hz following simulation are presented as frontal and lateral views in Figs.13 and 14. The distributions are presented by $d_\text {p}$ ranges of small (1–16 $\upmu$m), medium (18–26 $\upmu$m), and large (30–70 $\upmu$m) to better visualise deposition patterns.

For small particles, higher frequencies result in a broader dispersion and reduced anterior deposition, suggesting improved penetration. Medium-sized particles show a similar trend but with more pronounced deposition in posterior regions under higher frequencies. For large particles, deposition remains largely localised anteriorly, although some posterior transport is evident at 75 Hz. Comparatively, revision cases demonstrate more diffused and lateral deposition patterns, particularly under pulsating flow conditions, indicating that anatomical differences between Post-Op and Revision models influence particle transport and deposition.

## Discussion

This study investigated a T-junction geometry as a simplified analogue of the maxillary ostium to explore the impact of surgical modifications on particle transport and deposition. The T-junction serves as a representative model for branching flow pathways commonly found in both physiological and engineered systems. Specifically, the study examined how variations in anterior and posterior curvature radii ($R_{\text {c}}$) and pulsating flow frequencies influence particle behaviour, with the goal of informing sinus surgery strategies.

A key objective of (FESS) is to enlarge the maxillary ostium to improve ventilation and mucociliary clearance. (Anselmo-Lima et al. 2007; Hood et al. 2009; Xiong et al. 2008). While computational models have shown that ostium widening (i.e. diameter enlargement) reduces airflow resistance, the present study demonstrates that curvature adjustments ($R_{\text {c}}$) represent a distinct type of geometric modification that influences particle deposition through localised modification rather than global enlargement. Such modifications may reduce the likelihood of therapeutic particle deposition in targeted areas or, conversely, increase deposition in unintended regions due to altered flow dynamics (Frank et al. 2013; Xiong et al. 2008; Chen et al. 2011). These findings suggest that targeted curvature adjustments could optimise drug delivery while minimising the risks associated with extensive anatomical removal, such as mucosal drying or impaired clearance.

This work revealed that particle distribution, whether depositing on T-junction walls or exiting through the *x*- or *y*-branch, depends significantly on the location of the $R_{\text {c}}$ and the pulsation frequency magnitude. When the T-junction is considered as a simplified model of the maxillary ostium, these findings carry clinical relevance. Specifically, an anterior $R_{\text {c}}$ increased particle outflow through the *y*-branch and may lead to enhanced drug delivery to the maxillary ostium, whereas a posterior $R_{\text {c}}$ produced the opposite effect. Although both anterior and posterior $R_{\text {c}}$ configurations improved particle penetration, an anterior $R_{\text {c}}$ alone was more effective. This suggests that adjusting only the anterior $R_{\text {c}}$ of the T-junction, rather than increasing the overall diameter, may be an effective approach for enhancing particle penetration.

The location of curvature ($R_{\text {c}}$) plays a critical role in governing particle penetration into the *y*-branch. An anterior $R_{\text {c}}$, positioned upstream of the bifurcation, induces gradual flow redirection via centrifugal acceleration, increasing the likelihood of particles entering the lateral branch. In contrast, a posterior $R_{\text {c}}$ disrupts flow symmetry downstream, generating recirculation zones that hinder penetration and promote axial retention. These effects are particularly evident for particles with moderate inertia and are consistent with curvature-driven flow steering observed in other bifurcating systems.

An oscillatory boundary condition was applied to simulate pulsating flow delivery devices, such as those found in nebulisers and oscillatory drug delivery devices, which are designed to improve particle penetration into perpendicular bifurcations (Farnoud et al. 2020; Pradhan and Guha 2019). Although pulsating flow was often considered a method to enhance outflow through perpendicular pathways, this study found that better penetration occurred under a constant flow rate in the T-junction and decreased with increasing frequency. This outcome is consistent with the recent *in vitro* study by Seifelnasr et al. (2025), where VG/PG-based e-vape aerosols were shown to enter the maxillary sinus under a constant inhalation rate (2 L/min), driven by a sustained pressure gradient and two-way exchange flow across the ostium. In the current simulations, constant flow preserved axial momentum and promoted curvature-induced redirection, particularly with anterior $R_{\text {c}}$, allowing greater inertial penetration into the normal $y-$branch. These findings suggest that low, steady inhalation may be more effective for bulk sinus ventilation and particle delivery.

In contrast, pulsatile flow introduced time-varying velocity gradients, which generated oscillatory vortices and periodic flow reversal at the T-junction. While these effects increased particle residence time and enhanced deposition on the *y*-branch wall, they reduced net particle penetration into the outlet. The deposition enhancement was most pronounced for particles $\le$ 30 $\upmu$m with diminishing benefits at higher frequencies due to attenuation of velocity fluctuations near the ostium. These results imply that pulsation may be beneficial when targeted deposition at the sinus entrance is desired, but less effective for maxillary sinus deposition. Consequently, both flow condition and particle size distribution should be carefully matched to the clinical objective, whether it be ostium or sinus drug deposition.

A comparison of pulsating frequencies showed that 30 Hz was more suitable for particle penetration at the *y*-branch outlet and increasing the pulsating frequency had a negative effect. Although a pulsating frequency of 45 Hz is still considered the industry standard, for nasal nebuliser drug delivery, with several studies adopting it for investigations into pulsating drug delivery devices (Farnoud et al. 2021; Becker et al. 2022; Xi et al. 2017; Moeller et al. 2014, 2009; Laube 2007), our study suggests that lowering the frequency could result in higher drug deposition efficiency.

Recent research indicated that aligning the acoustic frequency with the patient’s sinus resonance frequency could significantly enhance deposition (Pourmehran et al. 2021, 2020; El Merhie et al. 2016). Interestingly, the higher applied frequency of 75 Hz did not result in additional outflow, possibly due to suction effects around the *y*-branch that forced particles back into the *x*-branch, leading them to either exit through the *x*-branch outlet or deposit on the wall.

The results obtained from the nasal cavity models were consistent with trends observed in the simplified T-junction geometry, demonstrating that pulsatile flow conditions significantly influence particle deposition. A pulsation frequency of 30 Hz led to increased particle deposition within the maxillary sinus in both the post-op and revision models. In the left maxillary sinus of the post-op model, deposition was observed exclusively under the 30 Hz condition, whereas in the right maxillary ostium, deposition occurred only under the 75 Hz boundary condition. These findings underscore the sensitivity of particle transport to pulsation frequency, as the imposed flow conditions substantially alter the flow field within the nasal cavity. Notably, previous investigations using the same nasal models showed that increasing the frequency to 75 Hz reduces the normal velocity fluctuations at the maxillary ostium, which may explain the frequency-dependent variation in deposition patterns (Warfield-McAlpine et al. 2025).

In the revision airway, particles within the range 15 < $d_\text {p}$ < 30 $\mu$m deposited in both left and right maxillary ostia across all assessed frequencies. This outcome reflects the aggressive surgical modification, which resulted in complete widening of the ostial openings. In contrast, the post-op model featured a substantially smaller ostium, yet particles in the range 15 < $d_\text {p}$ < 20 $\upmu$m were still able to penetrate the right maxillary sinus, despite their high inertia. To validate these findings, a benchtop “two-bottle” test rig incorporating an idealised T-junction under controlled pulsatile flow is recommended. Such experiments could provide direct visualisation of particle trajectories, verify CFD-predicted penetration, and support clinical translation of geometry-driven drug delivery strategies.

Both the revision and post-op models showed low deposition fractions ($\approx$ 1 $\times$ $10^{-3}$ and 1 $\times$ $10^{-4}$ for the left and right ostium, respectively); this is due to the lateral orientation of the maxillary sinus relative to the primary airflow path, repeated dosing in clinical settings (e.g. nebulisation) may achieve cumulative deposition sufficient for therapeutic effect. Exhalation dynamics were not modelled as clinical dosing occurs during inhalation only; Consequently, the results correlate to inspiratory transport and deposition.

It is important to note that this study kept the pulsation amplitude constant and did not include acoustic damping effects. As a result, potential resonance phenomena, such as those that may occur in Helmholtz-like systems, were not captured. This limits our understanding of how unsteady flow and geometry might interact under more realistic conditions. Future studies should explore variable amplitude inputs and include acoustic modelling to better assess how these factors influence on particle transport and deposition. These findings emphasise the need to consider both flow dynamics and acoustic effects when evaluating surgical outcomes and inhalation therapies.

Direct validation of the simplified T-junction cases was not feasible, as no comparable *in vitro* datasets currently exist for this idealised geometry. Instead, validation was undertaken in the patient-specific nasal models, which were compared against established experimental and numerical datasets (Kelly et al. 2004; Shi et al. 2008) and showed close agreement, Fig. 10. The T-junction was therefore used primarily as an analogue to isolate the effects of curvature and pulsation. Importantly, the deposition trends identified in the T-junction models were consistent with those observed in the anatomical nasal cavity models. This alignment provides indirect validation and supports the use of the T-junction as a proxy for understanding maxillary ostium transport phenomena.

This study provides insight into particle penetration through the maxillary ostium but is constrained by the use of simplified T-junction models and analysis of only a single patient-specific anatomy. The post-op and revision cases were not intended to provide population-level generalisability but rather to demonstrate how curvature and pulsation trends observed in the T-junction translate to anatomically realistic geometries. Given the substantial inter-individual variability in nasal anatomy, these findings should be interpreted as foundational, with broader applicability requiring future studies incorporating multiple patient geometries to establish clinical relevance.

## Conclusion

This research highlighted the significant impact of T-junction geometry modifications on particle distribution and deposition, with direct applications in drug delivery to the maxillary sinus. The findings demonstrate that modifying the anterior $R_{\text {c}}$ enhanced particle penetration through the *y*-branch more effectively than altering both anterior and posterior $R_{\text {c}}$. Moreover, a zero pulsating frequency promoted greater particle outflow through the *y*-branch. These findings offer new directions for surgical planning and the development of advanced patent specific inhalation therapies and flow conditions.

We found that:

- Anterior $R_{\text {c}}$ improves particle penetration more effectively than a posterior $R_{\text {c}}$ or a combination of anterior or posterior $R_{\text {c}}$.
- A constant flow condition (0 Hz) provides greater particle penetration through a normal flow outlet (*y*-branch outlet) in a T-junction model, but pulsation increases deposition on the *y*-branch wall.
- Deposition trends observed in the T-junction model aligned with results from anatomical nasal cavity models.
- Only small particles ($\le$ 30 $\upmu$m) showed deposition in the maxillary sinus under pulsating conditions for both nasal models.
- Simplified models provide a foundation, but broader anatomical variability and additional physiological factors must be integrated in future studies.

## Supplementary Information

Below is the link to the electronic supplementary material.

Supplementary file 1 (pdf 324 KB)

## Data Availability

The data that supports the findings of this study are available upon request.
