# Supplementary material for: Investigation of particle penetration in a nasal maxillary ostium with optimised T-junction geometry and pulsatile flow
Source: Biomech Model Mechanobiol. 2025 Oct 6;24(6):2217–35. doi: 10.1007/s10237-025-02015-9 (PMC12618317; doi:10.1007/s10237-025-02015-9)
Supplement: Supplementary file 1 — (pdf 324 KB) [file 10237_2025_2015_MOESM1_ESM.pdf]

Supplementary Material

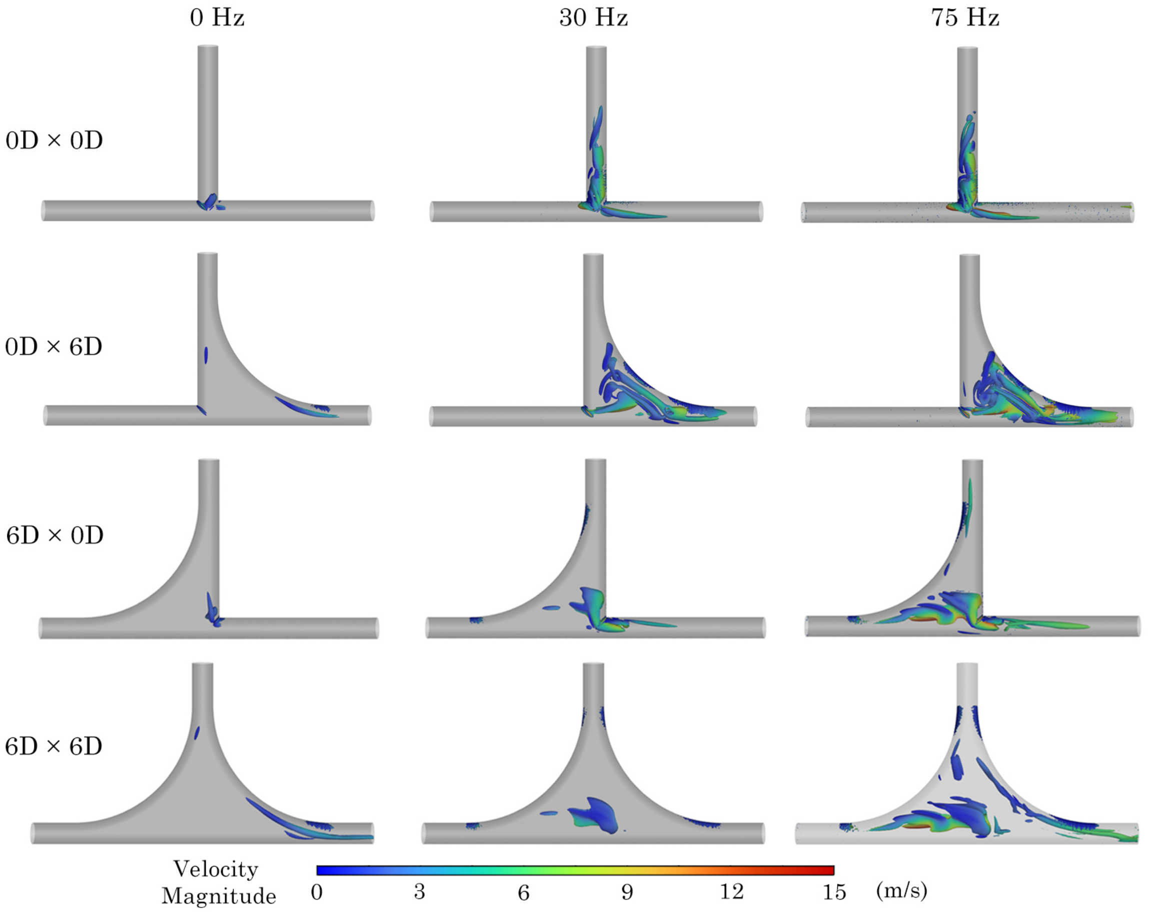

Figure S1: Iso-surfaces of Q-criterion ($Q = 6 \times 10^6\,\mathrm{s}^{-2}$) coloured by velocity magnitude (0-15 m/s) in the T-junction under steady flow (0 Hz) and pulsatile flow (30, 75 Hz) for four curvature configurations ($0D \times 0D$, $0D \times 6D$, $6D \times 0D$, $6D \times 6D$).

Fig. S1 illustrates Q-criterion iso-surfaces ($Q = 6 \times 10^6\,\mathrm{s}^{-2}$) for the T-junction configurations, showing the influence of pulsation frequency and R_c on vortex dynamics. Under steady inflow (0 Hz), vortical structures were weak and confined near the junction walls. At 30 Hz, coherent vortices formed in the y-branch, enhancing particle deposition. At 75 Hz, vortices become more intense and compact but remained closer to the junction, redirecting flow toward the axial branch with elevated near-wall velocities. Anterior ($0D \times 6D$) and combined ($6D \times 6D$) curvatures produced larger, more persistent vortices than symmetric ($0D \times 0D$) and posterior ($6D \times 0D$) cases, underscoring the role of local curvature in modulating pulsatile transport.
